# Disparities in the Demographic Composition of The Cancer Imaging Archive

**DOI:** 10.1148/rycan.230100

**Published:** 2024-01-19

**Authors:** Aidan Dulaney, John Virostko

**Affiliations:** From the Department of Diagnostic Medicine (A.D., J.V.), Livestrong Cancer Institutes (J.V.), and Department of Oncology (J.V.), Dell Medical School, University of Texas at Austin, 210 E 24th St, Austin, TX 78712; and Oden Institute for Computational Engineering and Sciences, University of Texas at Austin, Austin, Tex (J.V.).

**Keywords:** Ethics, Meta-Analysis, Health Disparities, Cancer Health Disparities, Machine Learning, Artificial Intelligence, Race, Ethnicity, Sex, Age, Bias

## Abstract

**Purpose:**

To characterize the demographic distribution of The Cancer Imaging Archive (TCIA) studies and compare them with those of the U.S. cancer population.

**Materials and Methods:**

In this retrospective study, data from TCIA studies were examined for the inclusion of demographic information. Of 189 studies in TCIA up until April 2023, a total of 83 human cancer studies were found to contain supporting demographic data. The median patient age and the sex, race, and ethnicity proportions of each study were calculated and compared with those of the U.S. cancer population, provided by the Surveillance, Epidemiology, and End Results Program and the Centers for Disease Control and Prevention U.S. Cancer Statistics Data Visualizations Tool.

**Results:**

The median age of TCIA patients was found to be 6.84 years lower than that of the U.S. cancer population (*P* = .047) and contained more female than male patients (53% vs 47%). American Indian and Alaska Native, Black or African American, and Hispanic patients were underrepresented in TCIA studies by 47.7%, 35.8%, and 14.7%, respectively, compared with the U.S. cancer population.

**Conclusion:**

The results demonstrate that the patient demographics of TCIA data sets do not reflect those of the U.S. cancer population, which may decrease the generalizability of artificial intelligence radiology tools developed using these imaging data sets.

**Keywords:** Ethics, Meta-Analysis, Health Disparities, Cancer Health Disparities, Machine Learning, Artificial Intelligence, Race, Ethnicity, Sex, Age, Bias

Published under a CC BY 4.0 license.

See also the commentary by Miles and Porras in this issue

## Graphical abstract

**Figure:**
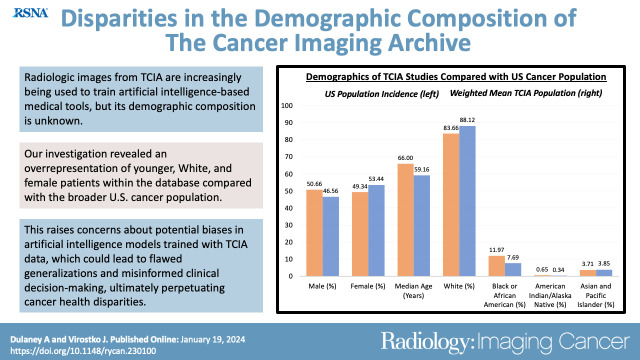

**Summary**

Patient demographics from data sets in The Cancer Imaging Archive did not reflect the demographics of the U.S. cancer population; thus, artificial intelligence tools developed using this database may perpetuate cancer health disparities.

**Key Points**

■ Demographic information was not provided for the majority of The Cancer Imaging Archive (TCIA) studies (available in only 83 of 189 studies), despite known influences of age, sex, race, and ethnicity on image features.
■ The median age of TCIA study patients was lower than the median age at cancer diagnosis in the U.S. population by more than 6 years.
■ American Indian and Alaska Native, Black or African American, and Hispanic patients were underrepresented in TCIA studies by 47.7%, 35.8%, and 14.7%, respectively.

## Introduction

Disparities in cancer incidence and mortality across race, ethnicity, and socioeconomic status may reflect health inequalities in the United States (1). Cancer health disparities result from a number of factors, including differences in health care coverage, socioeconomic status, exposure to risk factors, and genetic ancestry (2). Unfortunately, the biorepositories and data archives that help us better understand and treat cancer are largely composed of data from individuals with European ancestry (3). Inadequate representation of racial and ethnic minorities within these repositories hampers the generalizability of studies using them, thereby perpetuating cancer health disparities across these marginalized groups. For instance, the underrepresentation of Black patient populations in The Cancer Genome Atlas limits the ability to detect mutational frequencies in those populations (4). Furthermore, the scarcity of data from underrepresented patient populations obstructs our understanding of the complex relationship between race or ethnicity and cancer pathogenesis. There have been few large-scale studies, including a multiethnic cohort study (5), specifically targeting underrepresented groups to improve diversity in cancer research. Although initiatives to recruit diverse groups are in progress (6), these efforts are not yet well represented in existing cancer data sets that are being used to train artificial intelligence–based tools.

Radiology plays a crucial role in the screening, diagnosis, staging, and monitoring of cancer. The National Institutes of Health (NIH), recognizing the value of sharing de-identified medical images of cancer, funded The Cancer Imaging Archive (TCIA), which launched in 2011 (7) and, as of April 2023, hosts 189 imaging studies. TCIA image data sets have been employed in 1924 studies (as of April 2023; *https://www.cancerimagingarchive.net/publications/*). TCIA data are increasingly being used to train machine learning models (8,9). Indeed, TCIA tracking of keywords from publications using their data shows, as of April 2023, 438 publications performing “algorithm development,” 347 with the keyword “radiomics,” 287 performing “deep learning,” and 184 studies using “machine learning.” Thus, there is a pressing need to ensure that TCIA data are representative of those populations affected by cancer.

Medical images can be affected by a variety of demographic factors. Breast density is known to depend on age (10), which can in turn impact mammographic imaging. Race and ethnicity have been shown to influence both brain (11) and breast imaging (12). Aside from anatomic differences, sex can also influence tumor biology (13); thus, cancer imaging can be sex dependent (14). While the importance of population diversity in genetic studies is widely acknowledged (15), the rationale for including diverse patient populations in imaging studies is less established. However, it is essential that TCIA data sets reflect the actual demographic diversity of patients with cancer to encompass the full range of possible image features. Failure to do so may result in models trained on TCIA data that are inaccurate when applied to underrepresented populations. Indeed, it is well-known that the application of artificial intelligence in medicine can introduce biases against certain populations, leading to variations in performance on the basis of age, race, ethnicity, and/or sex (16). Previous reports have identified biases in the application of artificial intelligence to medical imaging. Models performing diagnosis from chest radiographs have been shown to underdiagnose underserved populations (17). Deep learning–based cardiac segmentation has similarly suffered because of disparities in the demographics of data used for training (18). Sex imbalance in medical images has introduced bias in classifiers used for computer-aided diagnosis of thoracic diseases (19). Given the widespread adoption of TCIA data for training radiologic machine learning models, any underlying disparities in TCIA demographics have the potential to generate biases that reinforce and perpetuate cancer health disparities. Therefore, in this study, we quantified the demographic composition of imaging data within TCIA, the largest publicly accessible repository of cancer imaging studies, to identify populations who are underrepresented compared with the overall U.S. cancer population.

## Materials and Methods

This retrospective study was exempt from institutional review board review because of the use of publicly available, de-identified data.

### Data Abstraction

**Figure 1:**
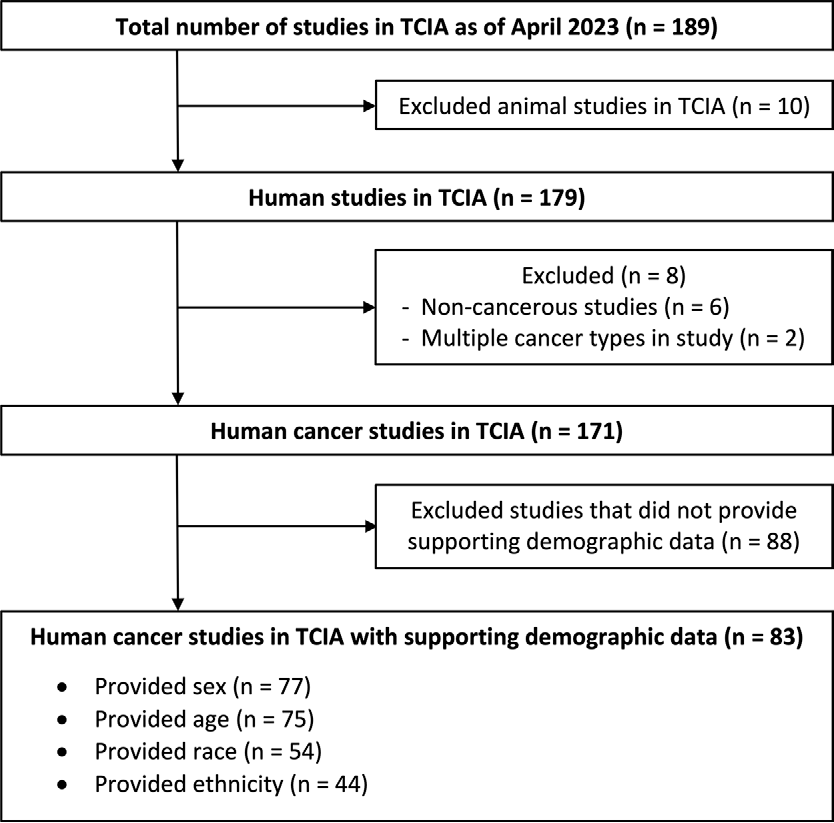
Flowchart of inclusion of studies for analysis. TCIA = The Cancer Imaging Archive.

The website for TCIA was accessed in April 2023 to generate a list of all studies hosted by the archive at that time (*n* = 189). TCIA organizes medical images into collections, which represent patient cohorts participating in specific imaging studies, typically related by primary tumor location. Each study was examined to determine the availability of demographic supporting data. In cases where demographic data were present, the mean, median, and SD in age were calculated, while proportions were determined for sex, race, and ethnicity. Among the initial 189 studies, a total of 106 were excluded as shown in Figure 1. These exclusions included 10 studies involving animal subjects, eight studies classified as noncancerous or encompassing various cancer types, and 88 studies that did not provide relevant demographic supporting data. Consequently, a total of 83 human cancer studies were identified to contain demographic data. Within this subset of 83 studies, demographic information regarding sex was available in 77 studies, age in 75 studies, race in 54 studies, and ethnicity in 44 studies.

### Data Analysis

The studies were sorted by primary tumor location. Race and ethnicity categories were adjusted by considering only the total number of known patients within each study, omitting any unknown or unreported data. Race categories were further standardized to align with the demographic classifications used by the NIH, which consist of White, Black or African American, American Indian and Alaska Native, and Asian and Pacific Islander. Ethnic categories captured by TCIA were Hispanic or Latino and Not Hispanic or Latino. To determine an average demographic statistic for each primary tumor location, a weighted average was calculated, weighting each individual study’s contribution on the basis of the number of patients in that specific study as a proportion of the total patients. The total cancer demographic categories were derived through a similar approach, weighting the average demographic statistics for each cancer type by the total number of patients.

### Cancer Population Demographic Information

The database of TCIA is composed primarily of studies performed in the United States. To obtain comparable demographic data reflecting the cancer population within the U.S. population, two sources were employed: the Surveillance, Epidemiology, and End Results (SEER) Program (20) and the Centers for Disease Control and Prevention (CDC) U.S. Cancer Statistics Data Visualizations Tool (21). The SEER Program was used to determine the median age at diagnosis for different cancer types, derived from the 2016–2020 incidence data. Using the CDC Data Visualizations Tool, the total number of new cancer cases from 2015 to 2019—categorized by sex, race, and ethnicity—was determined. Collectively, these data were used to establish comparable demographic information pertaining to age, sex, race, and ethnicity for the U.S. cancer population. For the three cancers with the most TCIA demographic data (breast cancer, lung cancer, and brain cancer), we examined heterogeneity in demographic information across individual studies. These subgroup analyses include the cancer with the highest incidence in the United States (breast cancer) and with the highest mortality (lung cancer).

### Statistical Analysis

The relative over- or underrepresentation of particular demographic groups was calculated by subtracting the U.S. cancer population of that group from the weighted TCIA proportion and dividing by the U.S. cancer population. Trends in demographic reporting over time were calculated using linear regression. Statistical analysis was performed using R (version 4.1.1; the R Foundation), with a *P* value of .05 considered as significant.

## Results

### All Cancers

**Figure 2:**
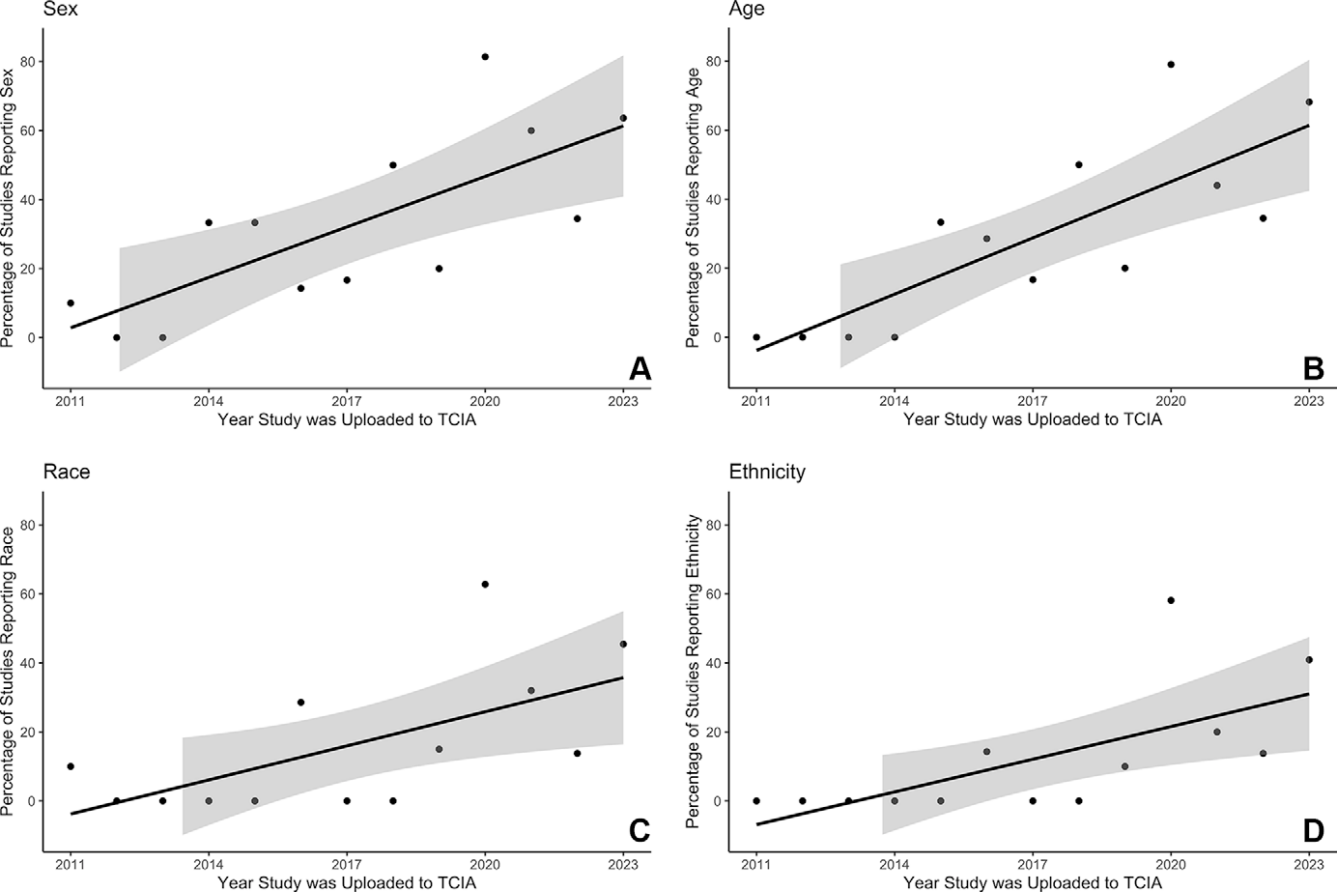
Scatterplots of TCIA study demographic reporting over time for **(A)** sex, **(B)** age, **(C)** race, and **(D)** ethnicity. Availability of all demographic information increased between 2011 and 2023. TCIA = The Cancer Imaging Archive.

Within the database of TCIA, information regarding sex, age, race, and ethnicity was found in a minority of human TCIA studies (Fig 1). Specifically, only 43% (77 of 179) of studies provided information on sex, 41.9% (75 of 179) on age, 30.2% (54 of 179) on race, and 24.6% (44 of 179) on ethnicity. Demographic information was present in 68.2% (15 of 22) of human breast cancer studies, 50% (13 of 26) of human lung cancer studies, and 61.1% (11 of 18) of human brain cancer studies, which were the three primary tumor subtypes with the largest number of studies. For breast, lung, and brain cancer studies, information regarding sex was available in 54.5% (12 of 22), 50% (13 of 26), and 61.1% (11 of 18) of studies, respectively; age in 54.5% (12 of 22), 42.3% (11 of 26), and 55.6% (10 of 18) of cases, respectively; race in 54.5% (12 of 22), 30.8% (eight of 26), and 33.3% (six of 18) of cases, respectively; and ethnicity in 36.4% (eight of 22), 23.1% (six of 26), and 27.8% (five of 18) of cases, respectively. We examined temporal trends in the availability of TCIA demographic data and found an increasing percentage of studies reporting demographic information over time. Significantly more studies reported sex (*P* = .003), age (*P* < .001), race (*P* = .022), and ethnicity (*P* =.012) from TCIA inception in 2011 through studies uploaded by April 2023 (Fig 2).

Across all TCIA studies analyzed (83 studies, 50 091 patients), female sex was overrepresented compared with the U.S. cancer population by 8.3% (Table 1). Breast, uterine, and ovarian cancer studies were well represented in TCIA, while prostate cancer studies were limited. Among tumor subtypes present in both sexes, female patients were most overrepresented in liver (16.9%), bladder (10.0%), and skin cancer (6.7%). Male patients were overrepresented in lung (15.6%), head and neck (14.6%), colorectal (10.6%), esophageal (8.1%), and pancreatic cancer (7.1%). Excluding tumor subtypes specific to sex-related anatomic features, thyroid, kidney, and brain cancers were closest to the U.S. cancer population by sex. The median age of TCIA patients was found to be 6.84 years lower than that of the U.S. population at the time of diagnosis. Lower median age in TCIA data than the general population at diagnosis was observed in breast (13.4 years), lung (10.6 years), pancreatic (7.7 years), and esophageal (7.0 years) cancer subtypes. Median patient age in skin (0.5 year), liver (0.84 year), colorectal (0.96 year), stomach (1 year), uterine (1.67 years), and brain (1.91 years) cancer subtypes was most similar to the age at diagnosis in the U.S. population. Across all cancer sites, White and Asian and Pacific Islander patients accounted for a higher proportion than the U.S. cancer population by 5.3% and 3.8%, respectively, while American Indian and Alaska Native and Black or African American patients were underrepresented by 47.7% and 35.8%, respectively. White patients were underrepresented in some cancer subtypes, including liver (31.4%), esophageal (21.3%), and bladder (9.0%) cancers. Asian and Pacific Islander patients were overrepresented in all cancer sites except for breast, prostate, uterine, and hematologic cancers. Black or African American patients were underrepresented for all cancers except for breast cancer. American Indian and Alaska Native patients were underrepresented for all subtypes other than colorectal and cervical cancer. Notably, no Black or African American or American Indian and Alaska Native patients were present in skin or hematologic cancer studies. The average proportion of Hispanic patients in TCIA studies was lower than that of the U.S. cancer population by 14.7%. Hispanic patients were underrepresented in all cancer subtypes except for head and neck and esophageal cancer. There were no skin cancer studies that reported Hispanic patients.

**Table 1:**
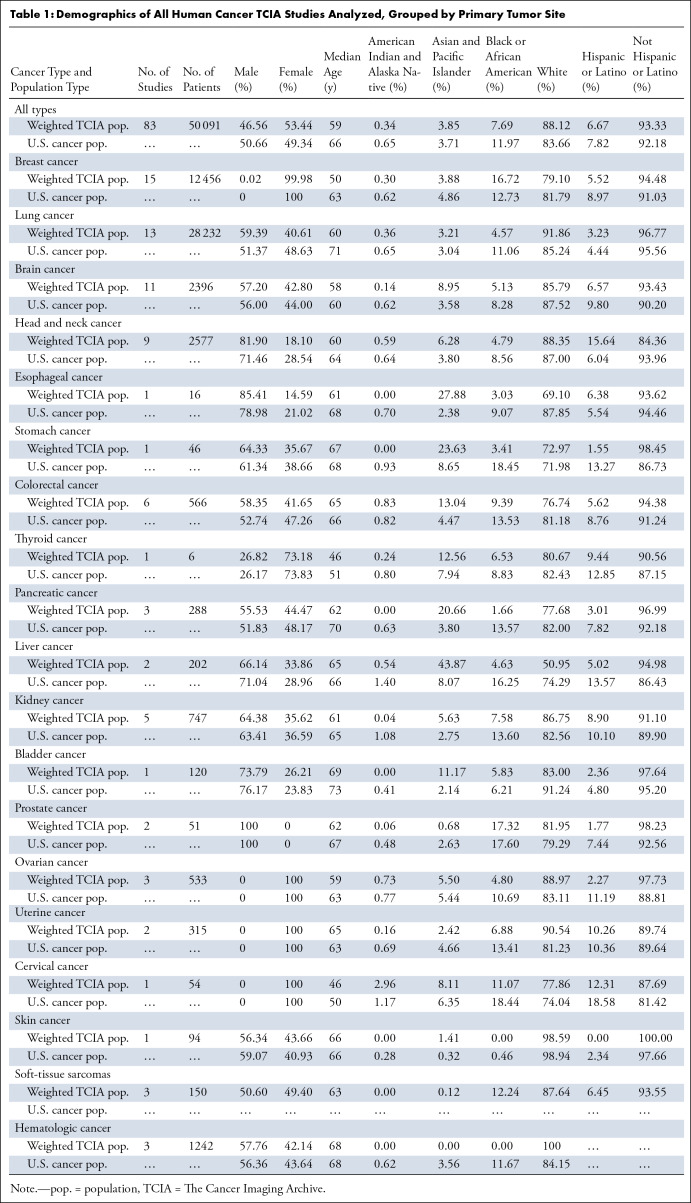
Demographics of All Human Cancer TCIA Studies Analyzed, Grouped by Primary Tumor Site

### Breast Cancer

The 15 TCIA studies reporting demographics of breast cancer (12 456 patients) showed heterogeneity across studies (Table 2). Only two breast cancer studies included male patients. Across all TCIA breast cancer studies, patients had a median age lower than that of the U.S. cancer population. On average, Black or African American patients were overrepresented by 31.3%, while White, American Indian and Alaska Native, and Asian and Pacific Islander patients were underrepresented by 3.3%, 51.6%, and 20.2%, respectively. Across all studies, White patients comprised 66.2%–92.2% of total patients, Black or African American patients comprised 4.9%–30.8%, American Indian and Alaska Native patients comprised 0%–15.8%, and Asian and Pacific Islander patients comprised 0%–12.3%. The average proportion of Hispanic patients was 38.5% lower than that of the U.S. cancer population. Hispanic underrepresentation was observed in all but two breast cancer studies.

**Table 2:**
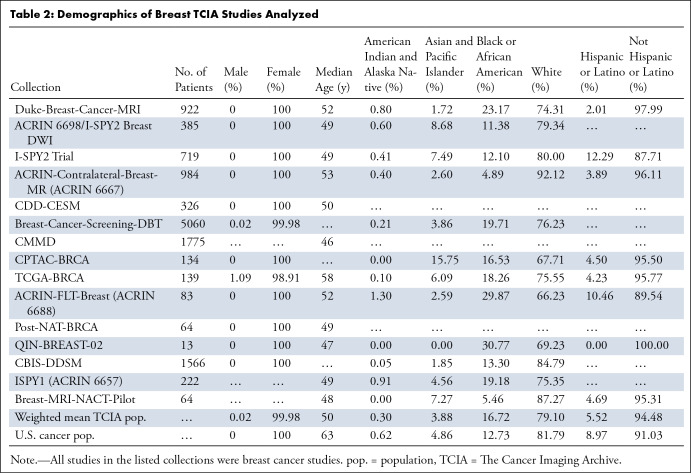
Demographics of Breast TCIA Studies Analyzed

### Lung Cancer

Lung cancer studies (13 studies, 28 232 patients) included the National Lung Screening Trial (ie, NLST), a large study comprising 93% of all patients from these studies, and therefore weighted heavily in the analysis shown in Table 3. Only one study exhibited a median patient age higher than the median age of the U.S. cancer population, resulting in an average age for all studies that was 10.6 years younger than that of the U.S. cancer population. Male sex was found to be overrepresented compared with the U.S. cancer population in all but one lung cancer study. On average, White and Asian and Pacific Islander patients were overrepresented by 7.8% and 5.6%, respectively, while Black or African American and American Indian and Alaska Native patients were underrepresented by 58.7% and 44.6%, respectively. Only two studies enrolled a greater percentage of Black or African American patients than that of the U.S. cancer population, and no studies enrolled more American Indian and Alaska Native patients compared with the U.S. cancer population. The average proportion of Hispanic patients was lower than that of the U.S. cancer population by 27.3%. Hispanic patients were underenrolled in all lung cancer studies.

**Table 3:**
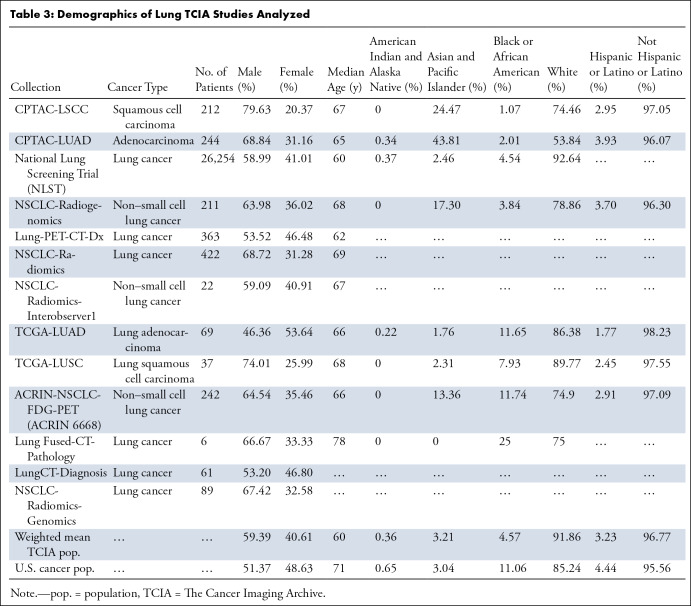
Demographics of Lung TCIA Studies Analyzed

### Brain Cancer

Across all brain cancer studies (11 studies, 2396 patients), male sex was overrepresented by 2.1% compared with the U.S. cancer population (Table 4). The median patient age in these studies was only 1.91 years younger than that of the U.S. cancer population. However, The Cancer Genome Atlas low-grade glioma (ie, TCGA-LGG) data set was an outlier, with a median patient age of 41 years, 19 years younger than that of the U.S. cancer population. On average, the proportion of White patients was lower than that of the U.S. cancer population by 2.0%. Black or African American and American Indian and Alaska Native patients were underrepresented by 38.0% and 77.4%, respectively. Interestingly, Asian and Pacific Islander patients were overrepresented by 150%, more than twice their representation in the U.S. incidence of brain cancer. This preponderance of Asian and Pacific Islander patients was driven largely by the Clinical Proteomic Tumor Analysis Consortium Glioblastoma Multiforme Discovery (ie, CPTAC-GBM) study, which exhibited the highest overrepresentation of Asian and Pacific Islander patients, with no Black or African American or American Indian and Alaska Native patients. The average proportion of Hispanic patients was lower than that of the U.S. cancer population by 33.0%, although two studies had greater than expected Hispanic representation.

**Table 4:**
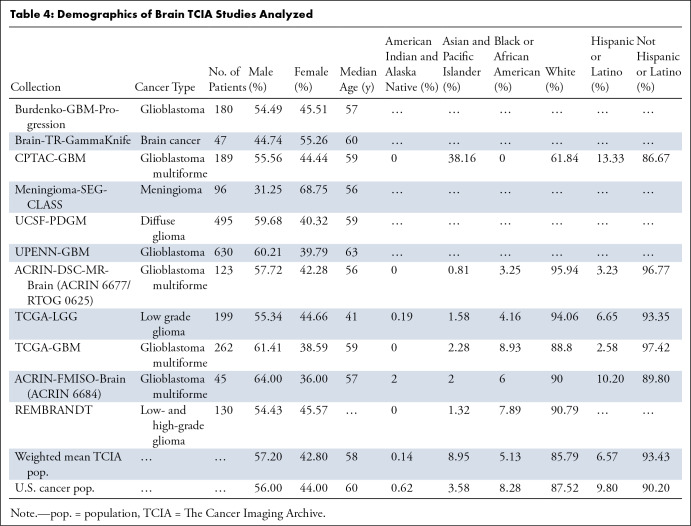
Demographics of Brain TCIA Studies Analyzed

## Discussion

In this study, we investigated the demographic composition of imaging studies within TCIA, the largest publicly available repository of cancer imaging studies, to determine whether they accurately reflect the demographics of the U.S. cancer population. Our results indicate that TCIA studies consist of younger patients and more females compared with the average U.S. population of patients with cancer. Additionally, our analysis revealed that Black or African American, American Indian and Alaska Native, and Hispanic patients were underrepresented across the vast majority of TCIA studies. Surprisingly, we found that the majority of TCIA studies do not provide demographic information for their patients. This absence of supporting demographic data hampers the analysis of the relationship between demographic factors and imaging features and limits the application of bias mitigation techniques that could enhance artificial intelligence tools trained using TCIA data.

We observed high heterogeneity in the level of ancillary data provided alongside medical images in TCIA studies. Less than half of TCIA studies included any demographic information, with racial and ethnic information provided by fewer than one-third and one-fourth of studies, respectively. Notably, many of these studies were performed using NIH funding, which requires the submission of Inclusion Enrollment Reports detailing race, ethnicity, and sex. This suggests that demographic data may have been collected for some TCIA studies but not uploaded along with their medical images. Contacting the current providers of TCIA studies and requesting demographic information, including any Inclusion Enrollment Reports already generated, could increase the proportion of demographic data sets within this database. Furthermore, future TCIA submissions could be prompted to provide supporting demographic information to improve the capture of these data during the study upload process. Of note, the current TCIA upload process includes a de-identification step with an option to retain sex, age, and ethnic group. Automatically opting-in studies to retain this demographic information may be one option to capture more of these data. Encouragingly, our temporal analysis found an increasing number of studies reporting demographic information over time, suggesting that TCIA is already improving the capture of demographic data.

We discovered that TCIA patients had a median age more than 6 years younger than the median age at cancer diagnosis in the United States. Among all primary tumor sites, only uterine and skin cancer TCIA studies had a higher median patient age than that of the U.S. cancer population at the time of diagnosis. The underrepresentation of older patients in cancer clinical trials has been a well-known issue for more than 2 decades and is believed to stem from patient and provider sentiments, study eligibility criteria, comorbidities, and logistic barriers (22). We found this discrepancy to be especially notable in breast cancer studies, considering the known changes in breast imaging with age (10). TCIA breast cancer studies had a median patient age that was 13 years younger than that of the U.S. population median age at diagnosis. Across all TCIA studies, more female than male patients were included. However, this likely reflects the high proportion of breast, uterine, ovarian, and cervical cancer images within the database of TCIA (27%), as opposed to the relatively minor contribution of prostate cancer images (0.1%). The underrepresentation of Black or African American, American Indian and Alaska Native, and Hispanic patients in the vast majority of TCIA studies aligns with their underrepresentation in National Cancer Institute clinical trials (23). One exception to this trend was observed in TCIA breast cancer studies where robust recruitment of Black or African American patients was evident. Strategies for increasing diversity in clinical trials have been identified (24); future cancer imaging studies should include these guidelines to expand outreach to underserved populations.

Our study had several limitations. Demographic information was available for less than half of the studies in TCIA. While we do not anticipate any systemic differences in demographics between the analyzed studies that provided demographic information and those that did not, this possibility cannot be excluded. Additionally, as a retrospective, single-database study, there may be inherent selection bias that could not be ruled out. Furthermore, we grouped together different imaging techniques to derive demographics across all studies, although artificial intelligence models would likely only include single modalities.

In conclusion, given the widespread use of TCIA data for training new radiologic machine learning models, it is crucial to characterize and address the disparities between the database of TCIA and the broader U.S. cancer population. These disparities introduce biases that can limit the data’s generalizability, misguide clinical decision-making, and overlook the nuances of patients’ health problems. Underrepresented demographic groups are particularly affected, leading to underdiagnosis in historically underserved populations (17). Radiologic artificial intelligence tools can perpetuate these inequalities by exacerbating health disparities among demographic groups who are inadequately represented in cancer imaging studies but whose health care continues to be influenced by the algorithms trained on such data. The inequitable TCIA representations identified and quantified in this study can be addressed and rectified. Moving forward, it is essential to prioritize the enrollment of a more diverse and equitable patient population in future cancer imaging studies. Furthermore, barriers to data sharing can hinder institutions from uploading data to TCIA, notably those serving patients in community settings. These challenges should be minimized to ensure equitable data sharing. However, for the studies already included in TCIA, our work focuses on addressing these structural inequities by quantifying them within each individual study. We advocate for the equitable weighting of the existing TCIA data through the implementation of bias mitigation strategies, such as stratified batch sampling or conducting independent analysis for each demographic group (25). These crucial steps aim to achieve a more accurate representation of the U.S. cancer population, thereby mitigating health disparities, enhancing the generalizability of developed tools, and ultimately, improving patient outcomes. By embracing these measures, we can provide patients with access to inclusive and individualized care through improved artificial intelligence tools for diagnosis and prognosis in medical treatment.
